# *NOF1* Encodes an Arabidopsis Protein Involved in the Control of rRNA Expression

**DOI:** 10.1371/journal.pone.0012829

**Published:** 2010-09-20

**Authors:** Erwana Harscoët, Bertrand Dubreucq, Jean-Christophe Palauqui, Loïc Lepiniec

**Affiliations:** INRA, IJPB, UMR 1318 INRA-AgroParisTech, Versailles, France; Umeå Plant Science Centre, Sweden

## Abstract

The control of ribosomal RNA biogenesis is essential for the regulation of protein synthesis in eukaryotic cells. Here, we report the characterization of *NOF1* that encodes a putative nucleolar protein involved in the control of rRNA expression in Arabidopsis. The gene has been isolated by T-DNA tagging and its function verified by the characterization of a second allele and genetic complementation of the mutants. The *nof1* mutants are affected in female gametogenesis and embryo development. This result is consistent with the detection of *NOF1* mRNA in all tissues throughout plant life's cycle, and preferentially in differentiating cells. Interestingly, the closely related proteins from zebra fish and yeast are also necessary for cell division and differentiation. We showed that the *nof1-1* mutant displays higher rRNA expression and hypomethylation of rRNA promoter. Taken together, the results presented here demonstrated that *NOF1* is an Arabidopsis gene involved in the control of rRNA expression, and suggested that it encodes a putative nucleolar protein, the function of which may be conserved in eukaryotes.

## Introduction

In order to identify genes involved in seed development or cellular housekeeping functions, several laboratories have used the model plant Arabidopsis for performing large-scale genetic screens [1], [2]. From these studies, the number of non-redundant genes essential for cell growth, division, and differentiation during gametophytes or/and seed development was estimated above 500 [3], [4]. Some of these genes have been shown to encodes for proteins that are involved in nucleolar functions [5], [6], [7], [8], [9], [10], [11]. The nucleolus is known to be involved in biogenesis of ribosome-subunits in eukaryotic cells [12], [13]. The initial ribosomal RNA (rRNA) precursor transcript is cleaved to form the mature 28S, 18S, and 5.85S rRNAs that are post-transcriptionally modified, through interactions with small nucleolar ribonucleoproteins (snoRNPs) [13], [14]. Then, with the help of other processing factors, rRNAs are assembled and exported into the cytoplasm. In Human, proteomic approaches have led to the identification of around 700 nucleolar proteins [15]. In plants, more than 200 nucleolar proteins have been identified [15], [16], [17]. A comparison of the nucleolar proteome from humans and yeast showed that 90% of human proteins have yeast homologues, thus demonstrating the strong conservation of nucleolar proteome through evolution [15]. However, plant and human nucleoli display some significant differences [16], [17], [18] and only 70% of the plant nucleolar proteins identified have human homologues (http://bioinf.scri.sari.ac.uk/cgi-bin/atnopdb/home).

Here, we report the isolation and functional characterization of *NOF1* that encodes for a nucleolar protein showing strong similarities with two proteins of yeast (YIL091C, accession n° Y21428) and zebra fish (DEF, accession n° Q6PEH4) [19]. The three conserved proteins appear to be necessary for the control of cell division or differentiation. Interestingly, the yeast protein interacts with several nucleolar proteins involved in rRNA biogenesis. In agreement with this function, we showed that the *nof1* mutants are affected in the methylation of rDNA promoter and rRNA expression.

## Results

### Isolation of the two allelic mutants *nof1-1* and *nof1-2* that are affected in embryo development

**Figure 1:**
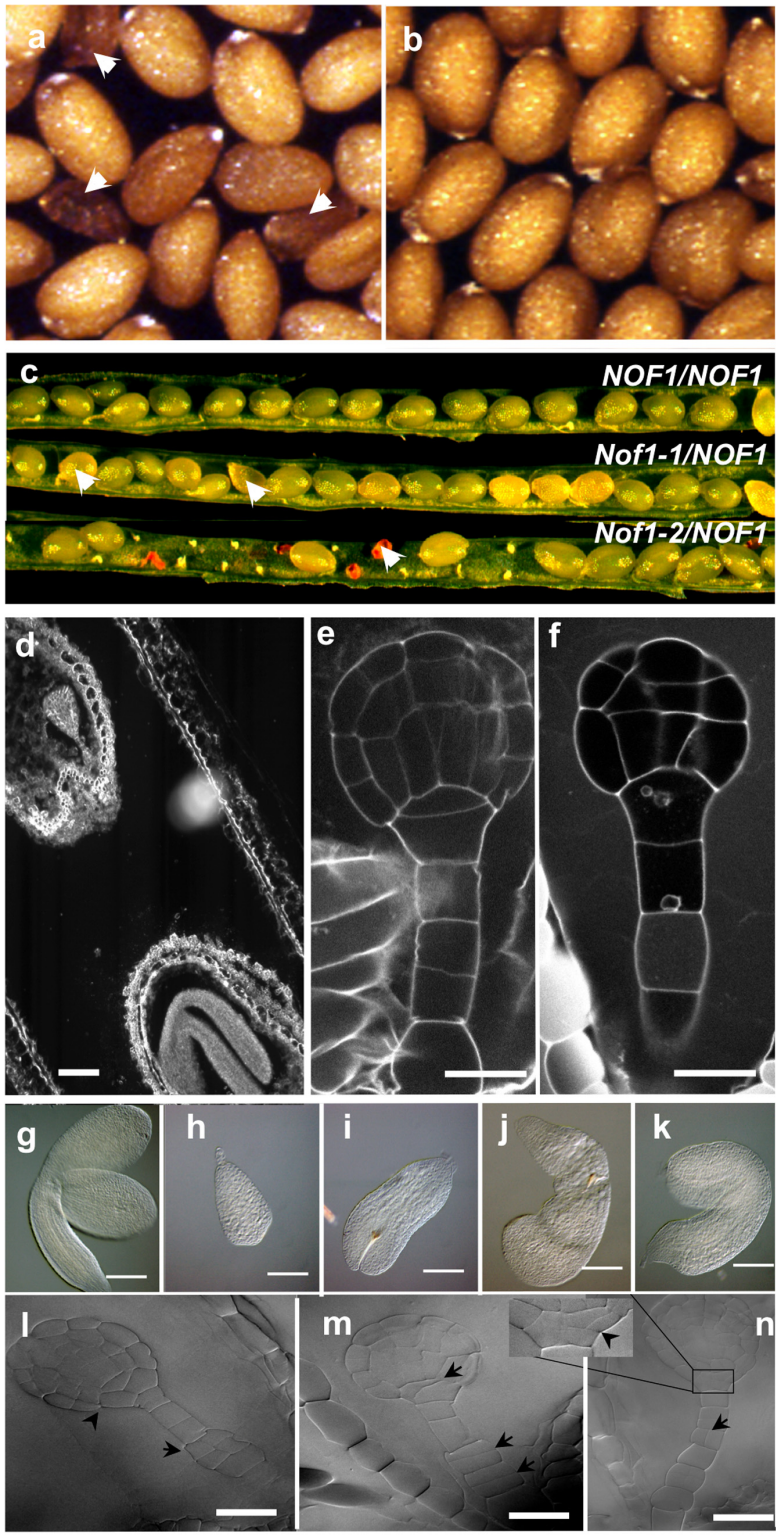
Phenotypic analyses of wild type and *nof1-1* mutant seeds. (a) Mature dry seeds from *nof1-1/NOF1-1* hemizygous plants displaying a few dark brown mutant seeds (indicated with arrows) and (b) Wild-type (Ws) control seeds. (c) representative developping siliques of wild-type accession (Ws) that displays immature green seeds (top row); *nof1-1/NOF1-1* (weak allele) genotype that display white (i.e. lethal) seeds (second row); and *nof1-2/NOF1-2 genotype* (third row) that display gaps (i.e. missing seed) and shrunken empty seed coat for the null allele *nof1-2*. (d) Late developmental stage in a single silique comparing a *nof1-1* mutant embryo (top left) to a wild type embryo (bottom right). Laser scanning confocal image of Ws embryo (e) compared to *nof1-1* globular embryo with abnormal cell divisions (f). Wild type embryo extracted from mature seed (g) compared to several *nof1-1* embryos arrested at different stages of development (h–k). DIC images of *nof1-1* embryos with abnormal cell divisions (arrows). Bars  = 600 µM (a, b, c), 10 µM (e, f, l, m, n), 100 µm (g–k).

A visual screening for abnormal seed morphologies of the Versailles' collection of T-DNA insertion lines was performed allowing the isolation of about 250 mutants [4]. One of these mutants, named *nucleolar factor 1-1 (nof1-1)*, was obtained in the progenies of the “DKE14” primary transformant. Plants hemizygous for the mutation appeared normal, except for the production of some wrinkled brown seeds (Figure 1a–c). Cytological analyses showed that the mutant seeds contain abnormal embryos, the development of which is arrested from early in the phase of pattern formation, to late in the maturation phase [20] (Figures 1g–k and S1). Although some of the developed *nof1-1* embryos were still metabolically active (Figure S1-B), the mutant seeds were unable to germinate. Consistent with this observation, the segregation analyses suggested that *nof1-1* mutation was recessive, monogenic, and lethal (Table S1). No homozygote plants for the mutation have been obtained. In addition, a strict co-segregation of the T-DNA (providing kanamycin resistance) with the abnormal wrinkled-seed phenotype was observed in the progenies of 110 plants. These results suggested the presence of a single T-DNA insertion locus in *nof1-1*, which was genetically linked to the mutation. Supporting this conclusion, DNA hybridization experiments with T-DNA specific probes showed that a single T-DNA was present in the mutant (data not shown). After the molecular identification of the *NOF1* gene, a second T-DNA insertion mutant named *nof1-2* was identified by reverse genetics screening in the progenies of the EXY42 primary transformant. No genetic complementation was observed when crossing two hemizygous lines (*NOF1*/*nof1-1* with *NOF1*/*nof1-2*), confirming that both mutants were allelic. Consistent with the embryo lethal phenotype of *nof1-1*, no homozygous plants were obtained with the *nof1-2* mutation.

### Gametes transmission is affected in the nof1 mutants

Segregation analyses showed that the number of seedlings resistant to kanamycin was significantly lower than expected for a dominant marker linked to a lethal mutation (63% for *nof1-1/NOF1* n = 2361 and 29% for *nof1-2/NOF1* n = 915, instead of 66,66%. Table S1). These segregations suggested that the mutated alleles, and more especially *nof1-2*, were transmitted to the progeny at a lower frequency than the wild-type allele. These results were consistent with the molecular analyses demonstrating that *nof1-2* is a null allele (see below) with abnormal ovule development (Figure 1c). Cytological analysis of cleared ovules showed that the putative *nof1-2* ovules were arrested during the mitotic divisions of the megagametogenesis (Figure S2). The reciprocal crosses between wild-type and hemizygous mutants lines confirmed that the transmission of *nof1-2* alleles was null through the female gametes and reduced through pollen (Table S2).

### The *nof1-1* mutant is affected in cellular division pattern

**Figure 2:**
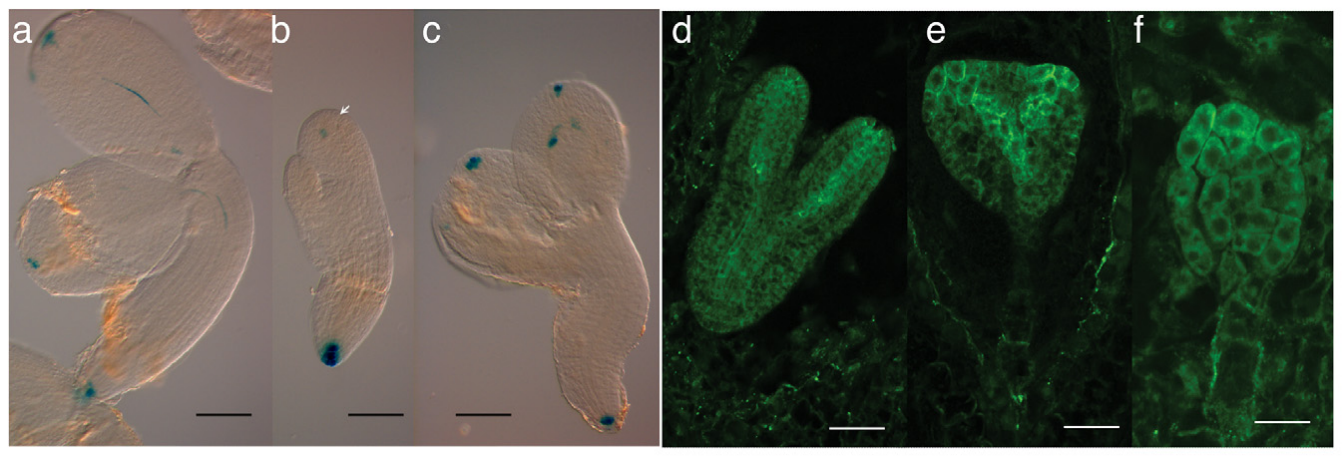
Auxin signaling in *nof1-1*. PRO*_DR5_*: uidA expression in wild type (a) and mutants (b and c) embryos. Immunolocalization of PIN1 in wild type (d) and *nof1-1* mutants (e and f) embryos. Bar  = 40 µm (a to c) and 100 µm (d to f).

In the wild-type developing embryo cells divide following a precise pattern [2]. Cytological observations revealed irregular pattern and/or additional cell divisions in *nof1-1* embryos (Figures 1d–f and S3A and B). The number of cell layers was sometime locally increased. In addition, lack of cell adhesion was found in embryos that are bent at the middle of the hypocotyl (Figure S3B). Although present, both meristems exhibited abnormalities such as a flat apical meristem or abnormal quiescent center in the root (Figure S3B). Taken together these data suggested that *nof1-1* embryos are affected in orientation and number of cell divisions. In addition, *NOF1* was preferentially expressed in differentiating cells (see hereafter and Figure 5A). In order to test if these phenotypes were associated with auxin signaling, the expressions of *pDR5*:*GUS* marker [21] and the localization of the auxin transporter PIN1 [22] were monitored in the mutant background. The reporter construct *pDR5*:*GUS* was introduced by crossing into *nof1-1* background and the localization of PIN1 was carried out by immunolocalization. In both cases, no striking differences were observed between the developed *nof1-1* and wild type embryos (Figure 2). Therefore, the abnormal *nof1-1* cellular phenotypes were probably not due to a default in auxin signaling. Nevertheless, they give an explanation to the abnormal development of *nof1-1* embryos.

### Isolation and molecular characterization of *NOF1*

**Figure 3:**
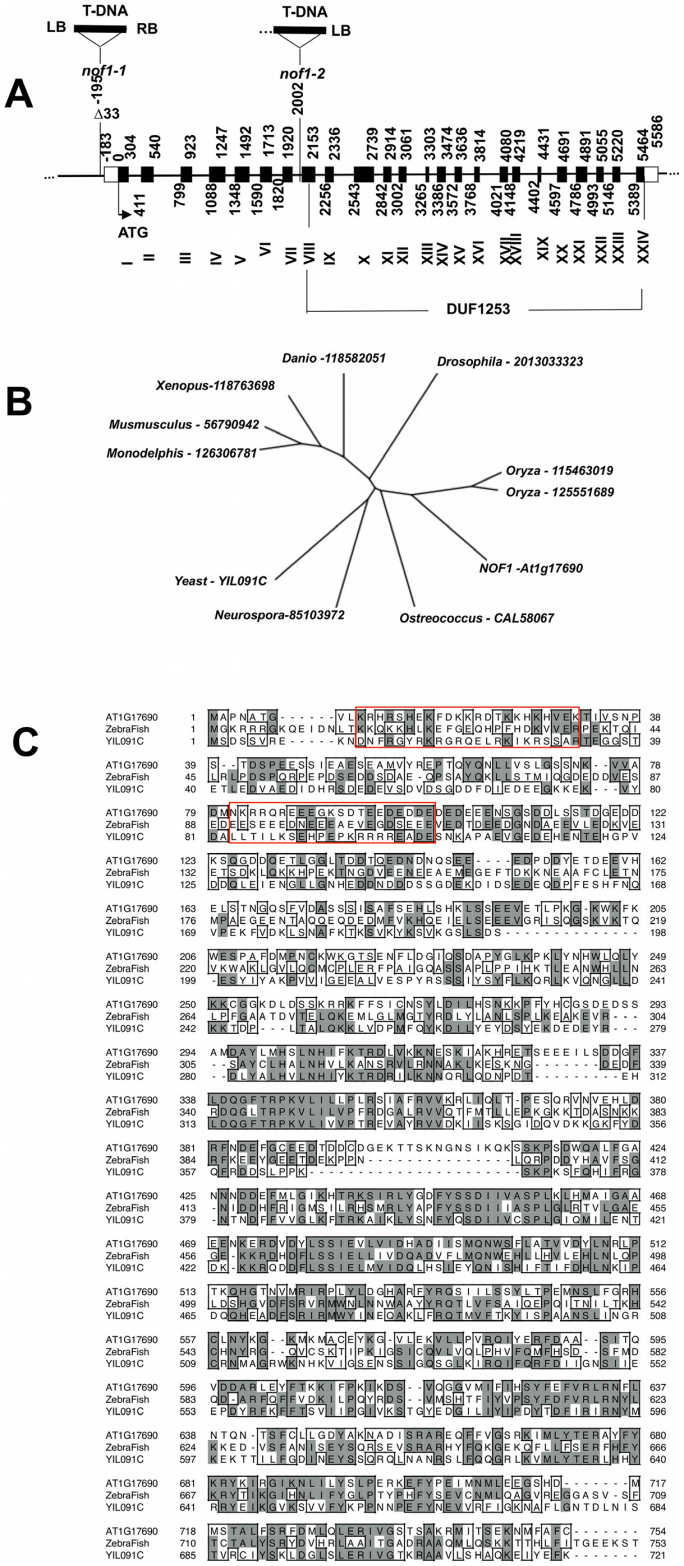
Molecular characterization of *NOF1*. **A**) Schematic representation of the structure of the *NOF1* (At1g17690) locus in wild type and *nof1-1* and *nof1-2* mutants. The structure of the gene is deduced from the comparison between genomic and cDNA sequences. In *nof 1-1*, the T-DNA was inserted between positions −195 to −228 relative to the ATG (first codon), leading to a small deletion of 33 bp. In *nof 1-2*, a T-DNA is inserted after nucleotide 2002. Boxes are exons. The 5′UTR is predicted according to ESTs sequences. Location of the DUF1253 domain is indicated below the scheme. **B**) Unrooted neighbour-Joining tree representing the distance between NOF1 and the most closely related proteins from various organisms was obtained using the full amino-acid sequences, after clustal W alignment (http://align.genome.jp/). **C**) Deduced amino acid sequences of NOF1 and closely related proteins are presented (At1g17690: NOF1, Zebrafish (DEF): gi37046654, and yeast YIL091C). Identities between amino acid residues are shown with dark boxes and similarities with light boxes. The putative NoLS site is boxed.

Isolation of the putative *NOF1* gene was performed using the T-DNA tagged *nof1-1* allele. Plant genomic sequences flanking the left (68 bp) and right (73 bp) T-DNA borders were recovered by walking PCR and sequenced [23]. The site of integration was assigned to chromosome 1 in the intergenic region at 195 bp upstream the ATG initiation codon of At1g17690 (Figure 3A). A small deletion of 33 bp was found at the insertion locus. A second allele was isolated by reverse genetic using FlagDB [24]. Localization of the T-DNA insertion (7 kb) in the gene suggested that *nof1-2* is likely a null allele (Figure 3A). In order to confirm the identity of *NOF1*, the genetic complementation of both alleles was obtained with a wild-type genomic clone (see material and methods and Figure S4). Taken together these data showed that *NOF1* is At1g17960. This gene encodes a putative protein of 754 amino-acid residues of unknown function. Sequencing of a cDNA and complementation of the *nof1-1* mutant with this cDNA fused to GFP confirmed this prediction (see next paragraph). The protein contains a potential nucleolar localization signal (NoLS) [25] and a conserved “DUF1253” domain of unknown function (Figure 3A). Only one *NOF1* gene is found in Arabidopsis and closely related genes were found in other eukaryotes (Figure 3B). For instance, DEF from danio [19] and YIL091C from yeast display about 50% identities at the amino-acid level (Figure 3C). This may indicated a conserved function among eukaryotes.

### *NOF1* is expressed in all tissues, but preferentially in differentiating cells

**Figure 4:**
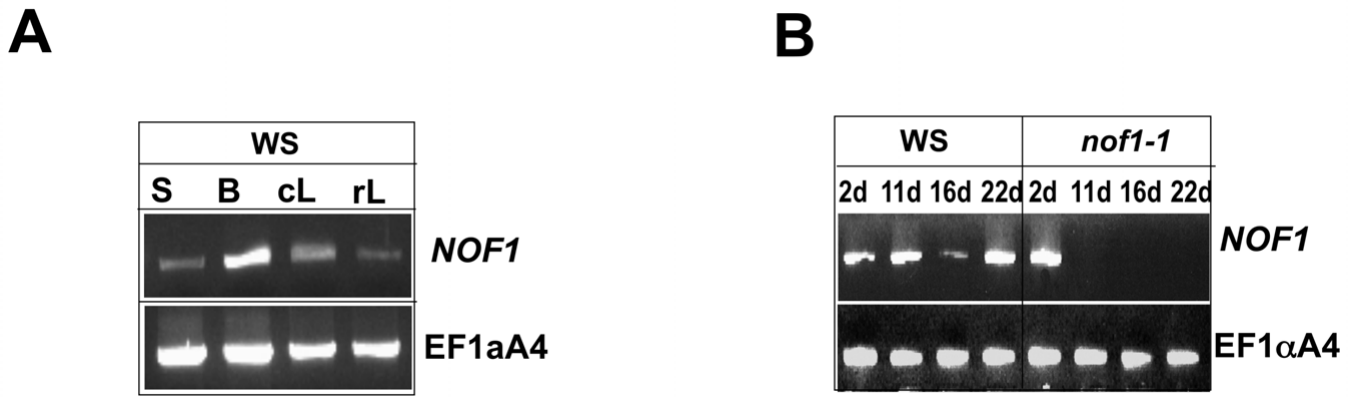
Analyses of *NOF1* mRNA accumulation in wild type and *nof1-1*. RNA was extracted from various plant organs (**A**) and seeds (**B**) at different stages of development and used for reverse transcription. Primers specific for *NOF1* and for *EF1αA4* as control were used on the same set of first strand cDNA templates generated with dT primers. During silique development (**B**), WS or *nof1-1* seeds were manually dissected based on seed phenotype to produce the *NOF1* cDNA template at 2, 11, 16 and 22 days after fertilization. S: seeds, B: buds, cL: cauline leaves, rL: rosette leaves.

**Figure 5:**
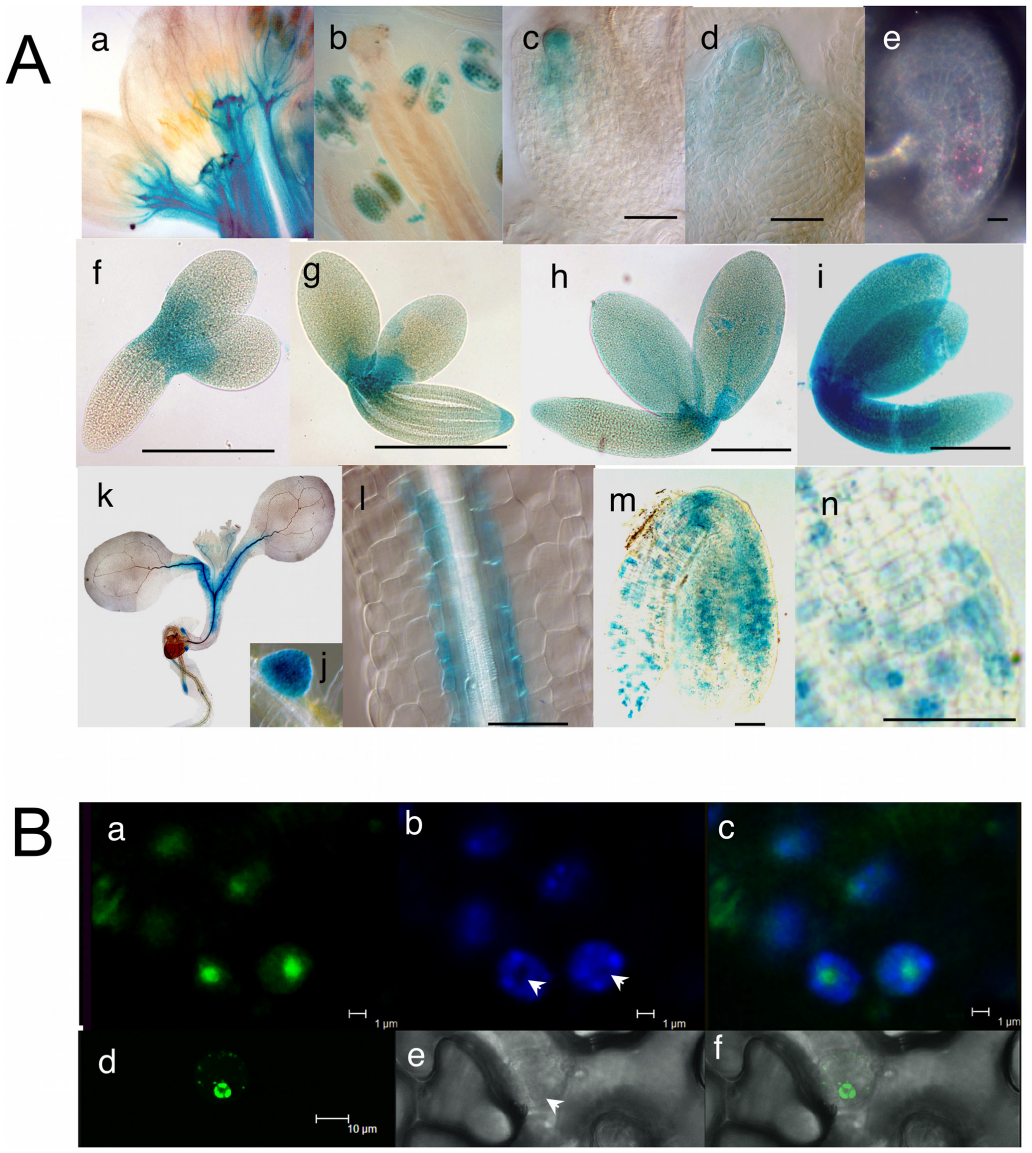
Cytological analyses of *NOF1* expression and intracellular localization of the protein. **A** The activity of the *NOF1* promoter (*pNOF*:*UidA*) was investigated in various plant organs. The result of GUS activity was observed with Nomarski optics, except for (e) where dark field is used, on flowers (a–b) showing expression in buds and in pollen. Expression was found in the nucellus of developing ovules (c–d) and later in the embryo sac (e). During embryogenesis, GUS activity is found at the top of the hypocotyl and extends throughout the embryo during maturation (f to i). After 24 h of imbibition, the expression is found with a patchy pattern in the embryo (l to n) before disappearing. Five days after imbibition's start, the expression is found in root apex (k), lateral root initials (j) and around vascular bundles (l). Bars  = 10 µm (c to e), 100 µm (f to i) and 50 µm (l to n). **B**). Subcellular localization of NOF1:GFP in transgenic Arabidopsis lines expressing *35S*:*NOF1*:*GFP*. GFP is detected in the nucleoli (a), DAPI staining (b) and merged image (c). Transient expression in tobacco leaves of *35S*:*NOF1*:*GFP* showing nucleolar localization of NOF1:GFP (d), transmitted light (e), and merged pictures (f). Bar  = 1 µm (a, b, c) and 10 µm (d, e, f).

The expression of *NOF1* was investigated in various tissues by RT-PCR. *NOF1* mRNA was detected in all organs tested (Figure 4A). These results were fully consistent with transcriptomic data available (Figure S5). Interestingly, in the weak mutant allele (*nof1-1*), some *NOF1* mRNA was still detected at 2 days after pollination (Figure 4B), probably due to the insertion of the T-DNA in the promoter region. This result was consistent with the weak phenotype of *nof1-1* compared to that of the null *nof1-2* (the latter does not produce embryos). However, it is difficult to fully avoid the hypothesis of a contamination by mRNA from the carpel. The spatio-temporal activity of the *pNOF1* promoter was then investigated in transgenic plant that express the *pNOF1*:*GUS* reporter construct. GUS activity was mainly detected in cells that undergo cellular differentiation in young tissues such as floral buds, ovules, embryo, secondary roots, pollen, young seedlings and vascular bundles (Figure 5A).

### *NOF1* is a nucleolar protein

In order to investigate intracellular localization of NOF1, a *NOF1*:*GFP* chimeric gene was introduced in a *nof1-1* hemizygous plant. Primary transformants exhibiting complementation of the *nof1-1* phenotype in their progenies were obtained, indicating that the chimeric construct was functional. By comparison with DAPI staining of the nucleus, a nucleolar localization of GFP was observed in young developing ovules (Figure 5B). No GFP was detected in other tissues suggesting a weak stability of the chimeric protein. The *NOF1*:*GFP* chimeric gene was transiently expressed in tobacco cells giving a similar result, with a preferential accumulation in the nucleolus (Figure 5B).

**Figure 6:**
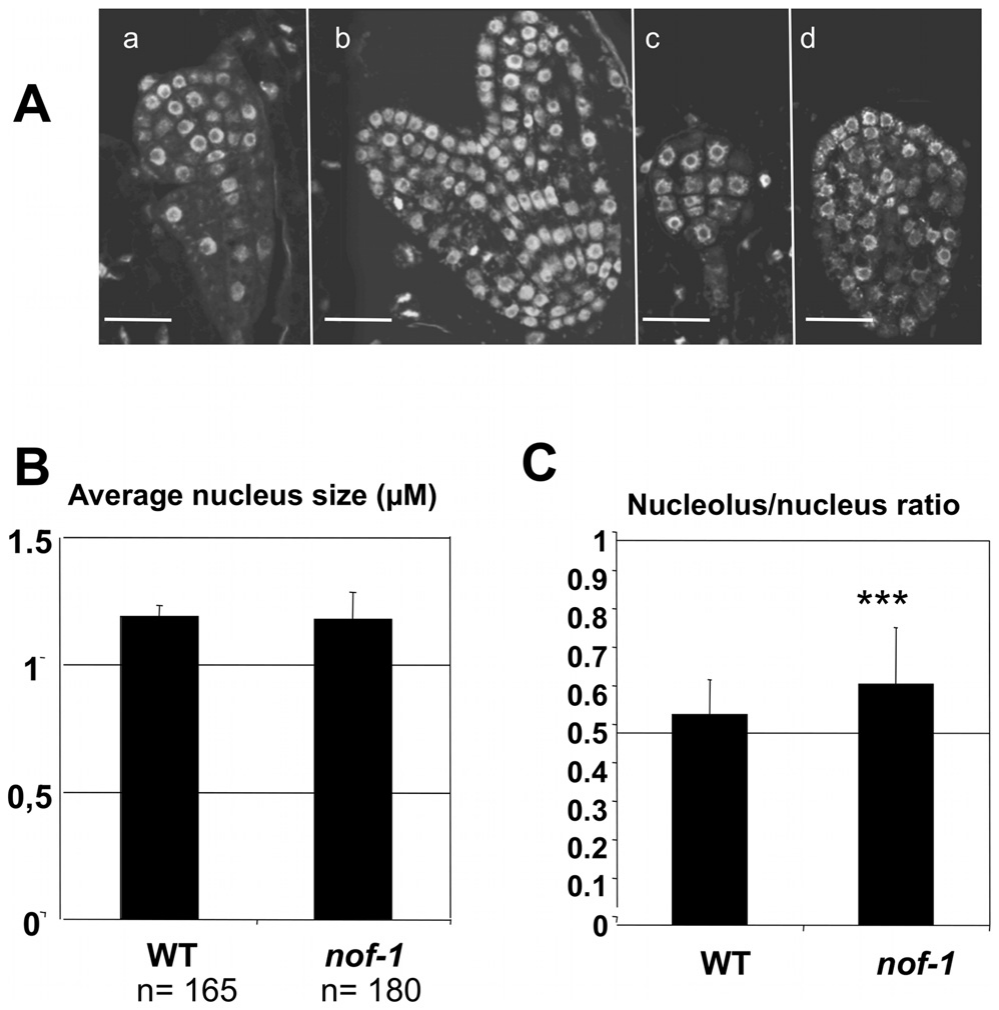
Nucleolus phenotypes. **A**) Embryo development at the globular and heart stage of development, Ws (a and b respectively) and *nof*1-1 (c and d respectively) after DAPI staining and laser confocal imaging. Bar  = 5 µm. **B**) Average nucleus diameter was measured in Ws and *nof1-1* embryos after DAPI staining and laser scanning confocal imaging. **C**) Average ratio of nucleolus *vs* nucleus diameters in Ws compared to *nof1-1*. A student test was performed to compare both populations of nucleoli, demonstrating a significant difference with p<0.0001 (t = −6.26).

It is known that modifications of nucleolar functions can affect the size of the nucleolus [10]. Therefore, a cytological analysis of the nucleus was made. Although no obvious differences were observed earlier in nucleolus morphology, the *nof1-1* cells showed enlarged nucleoli at the globular and heart stages of embryo development (Figure 6A). Image analyses, at the globular stage of embryo development, confirmed that although the size of the nuclei is not affected in *nof1-1* (Figure 6B), the nucleoli are significantly enlarged (Figure 6C). These data argue in favor of an important role for NOF1 in the nucleolus.

### The accumulation of *rRNA* increases in *nof1-1*

The yeast structural ortholog of NOF1 (i.e. YIL091C) has been recently shown to interact with nucleolar proteins MPP10 and SAS10 [26]. MPP10 and SAS10 are members of the small subunit of the rRNA processome (SSU) [27], [28], [29], [30]. These data are fully consistent with the nucleolar localization of NOF1 and the abnormal phenotype of the nucleoli in *nof-1-1* cells. Furthemore in silico analysis of genes co-expressed with *NOF1* revealed a strong bias for ribosome function as compared with randomly generated lists of genes (Figure S6).

**Figure 7:**
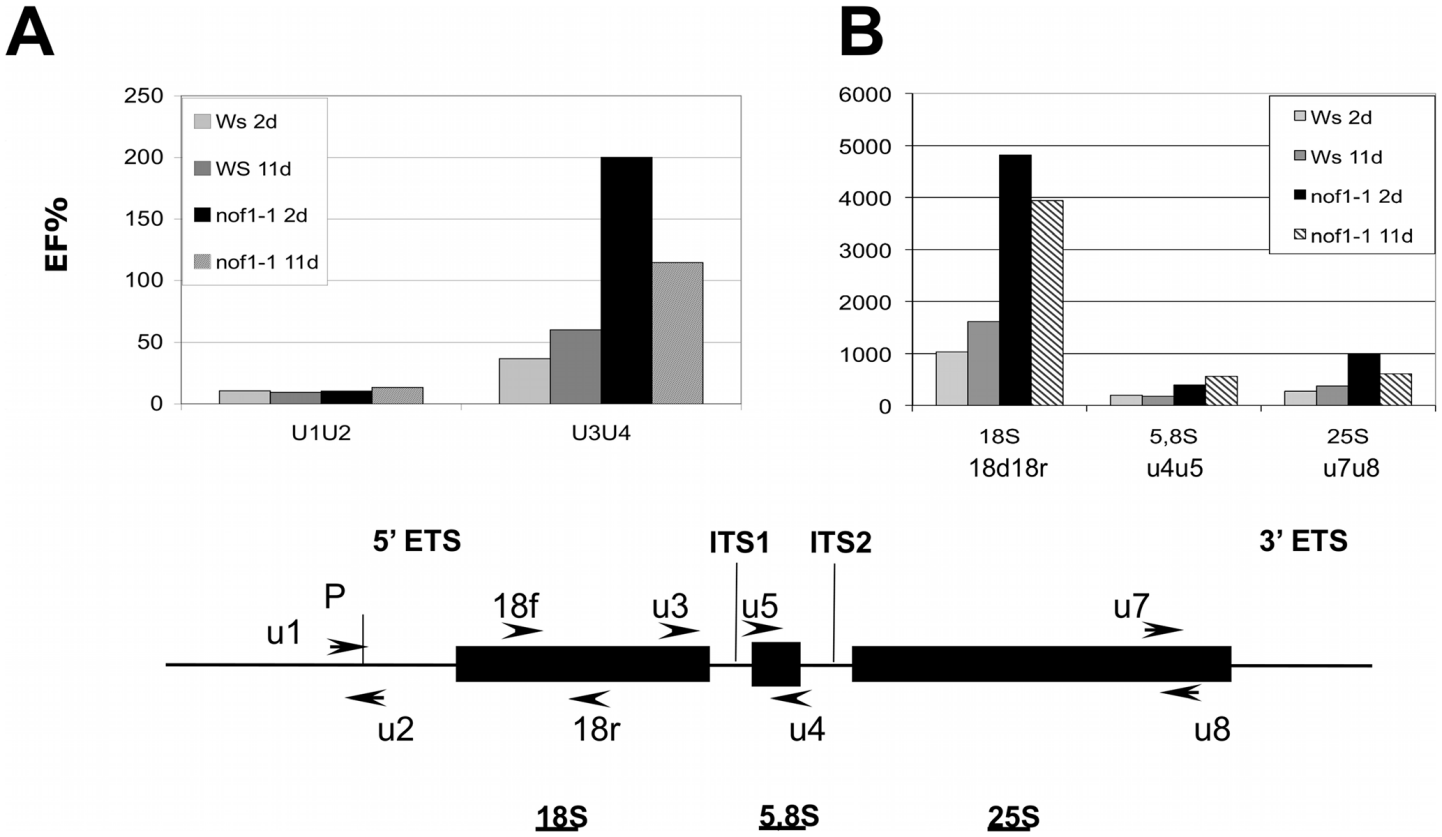
Expression and processing of the 45S rRNA. Accumulation of rRNA was monitored by qRT-PCR using specific primers for unprocessed (U1/U2, U3/U4) (**A**) and processed forms (i.e. 18S f/r, U5/U4 for 5.8S and U7/U8 for 25S) (**B**) of the 45S rRNA transcript. The cDNA templates were been obtained after manual seeds dissection from a hemizygous plants for the *nof1-1* mutation or wild-type (Ws), at 2 or 11 days after fertilization. One representative experiment of three independent biological repeat is shown.

Processing of the pre-rRNA is conserved among eukaryotes and has been described in details [5], [12], [14], [31], [32], [33]. The pre-rRNA is firstly cleaved at the P site located in the 5′ external transcript spacer (ETS) and then in the internal transcript spacers (ITS) (Figure 7). To confirm the involvement of NOF1 in rRNA biogenesis, the levels of pre-rRNA and mature 18S, 5.8S, and 25S rRNAs were monitored by quantitative RT-PCR (Figure 7). RNAs were extracted from mutant embryos obtained from white seeds collected in developing siliques at 2 and 11 days after pollination. In *nof1-1*, a strong increase in pre-rRNA accumulation was observed (Figure 7A). Similar increases were found for total rRNAs (Figure 7B). These results showed that the *nof1-1* mutation triggered a strong increase in rRNA expression.

### The methylation of rRNA promoter is affected in *nof1-1*

**Figure 8:**
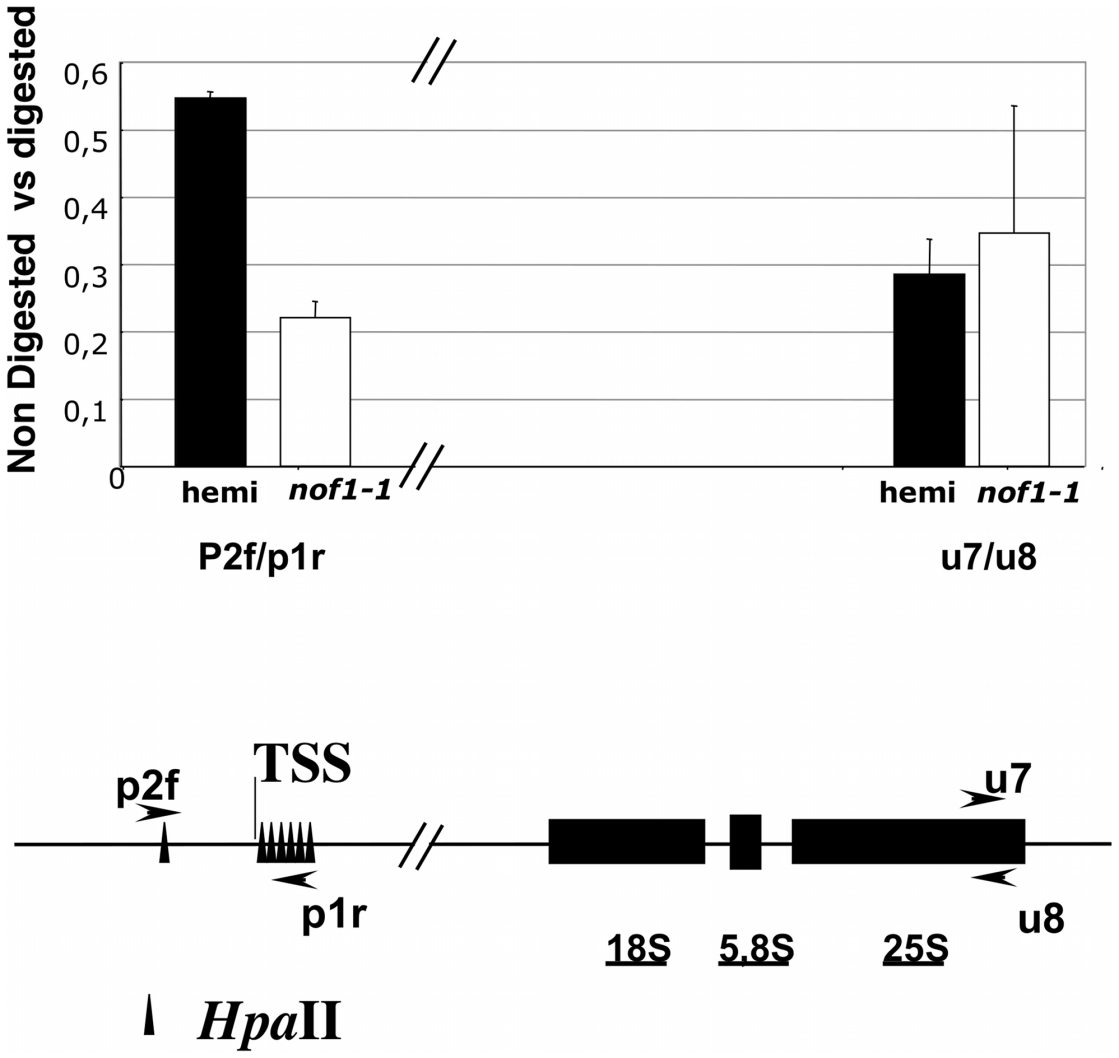
Quantification of DNA methylation at the 45S rDNA locus. The level of methylation of the promoter region was estimated by qPCR. Genomic DNA was PCR amplified directly or after restriction with a methylation sensitive enzyme (*Hpa*II) using specific primers (p2f and p2r). The amplification of the 25 s rDNA was used as internal control. Results are the means of 3 measurements (+/− standard deviation). Experiments have been made on two independent biological replicates showing similar results. TSS: transcription start site.

In plants, rDNA transcription is regulated by methylation of the promoter region [34], [35]. In Arabidopsis, the transcription start site (TSS) of the Polymerase I was shown to be methylation sensitive and to contain specific elements of regulation [36]. Interestingly, it has been recently shown that YIL091C, the putative yeast homolog of NOF1, interacts with JHD2, an histone (H3K4) demethylase [37], [38]. The methylation of histone 3 lysine 4 is an epigenetic mark that triggers DNA hypomethylation and thus increases transcription [39]. Therefore, it is tempting to speculate that the strong expression of rRNA in *nof1-1* could be due to the hypomethylation of rDNA promoter. To test this hypothesis, the level of methylation of rDNA promoter was investigated by quantitative RT-PCR after restriction of genomic DNA with a methylation sensitive enzyme (*Hpa*II). Amplifications were performed with TSS specific primers on DNA templates extracted from *nof1-1* dissected seeds, at 11 days after pollination (Figure 8). This method was more suitable than conventional bisulfite conversion for the limited amount of biological materials available after seed dissection and produced reproducible results [40], [41]. The rDNA promoters were significantly less methylated in *nof1-1* than in hemizygous embryos (Figure 8). In addition, the methylation of 25S rDNA region used as control was not affected in *nof1-1*. These results suggested that rDNA promoter is specifically hypomethylated in *nof1-1* that is consistent with higher expression of rRNA.

## Discussion

### *NOF1* is required for embryogenesis and gametogenesis in Arabidopsis

In this study, we have shown that NOF1 is essential for female gametogenesis and embryogenesis. The leaky allele *nof1-1*, that accumulates *NOF1* mRNA until a few days after fertilization, produced embryos exhibiting a broad range of abnormal cellular phenotypes (e.g. cell division pattern or lack of cell adhesion). These data are consistent with the expression data demonstrating that *NOF1* is expressed in all tissues, and preferentially in dividing cells. Since auxin is an important hormone in embryo development [42], [43], [44], [45], [46], we have investigated whether it may be involved in these abnormal cellular phenotypes. The immunolocalization of the PIN1 auxin transporter as well as the use of Pro_DR5_:GUS construct in the *nof1-1* mutant background did not reveal any obvious changes in auxin transport and accumulation. These results indicated that the abnormal cellular phenotypes are very likely not the consequence of a modified auxin accumulation or transport. The strongest *nof1* allele is impaired very early during female gametogenesis and the male gametogenesis is slightly affected. This result is fully consistent with the finding that several proteins involved in general cell cycle progression, including nucleolar proteins such as *SLOW WALKER 1* (*SWA1*, At2g47990) [5], play essential roles during female gametogenesis. Nevertheless, we cannot rule out the possibility that a redundant function may exist in pollen. In addition, although *NOF1* mRNA was found in all tissues, we do not know if NOF1 is really necessary for cell viability in all tissues during the entire plant life cycle. To answer these questions it would be interesting to build an inducible dominant negative system (e.g. RNAi) to switch off *NOF1* expression in specific tissues and cells.

### NOF1 is involved in nucleolar functions

Sequence analysis of the predicted NOF1 protein revealed potential nucleolus localization signal (NoLS) [47] in the N terminal part of the protein. In addition, the yeast ortholog (i.e. YIL091C) has been recently shown to interact with several nucleolar proteins [26]. The functional data reported here, both in tobacco and in Arabidopsis are consistent with a nucleolar localization of NOF1. Interestingly, despite the complementation of *nof1-1* abnormal phenotype by the ectopic expression of *NOF1*:*GFP* construct, GFP signal was detected only in the integuments of developing ovules. This result suggested a post-translational control of NOF1 in vegetative parts. A putative sumoylation site on lysine (K205) was also predicted using SUMOsp program [48]. Sumoylation is a reversible post-translational modification that appears to play a crucial role in a variety of biological processes [49]. It would be interesting to investigate if the putative sumoylation site is involved in NOF1 functions.

Consistent with the putative nucleolar localization of NOF1, its mutation affects the size of the nucleoli. A similar increase was previously reported in other embryo lethal mutants [8], [10]. In both cases, the mutated gene were directly linked to nucleolar functions and ribosome biogenesis. On this line, a link between rDNA transcription and the size of the nucleolus was recently reported using inhibitors of DNA methyl transferases [50]. Taken together these data showed that *NOF1* encodes a putative nucleolar protein, the function of which is important for the nucleolus, in agreement with its involvement in rRNA biogenesis.

### NOF1 is involved in the control of rRNA expression

In *nof1-1*, an increase in rRNA expression was observed, in association with hypomethylation of the rDNA promoter region. These genetic analyses support the view that NOF1 acts through rDNA transcription and is fully consistent with the demonstration that rDNA methylation negatively impacts rDNA transcription by polymerase I [34], [35], [36], [51], [52], [53], [54]. As NOF1, the closely related yeast (YIL091C) and danio (DEF) proteins are localized in the nucleus and are essential for cell viability [19], [55] suggesting a functional conservation of these proteins. Furthermore, DEF was shown to affects cellular differentiation and division as *def* mutants exhibit an arrest of expansion growth of digestive organs [19]. Interestingly, in yeast, YIL091C interacts with a H3K4 demethylase [37], [38] and demethylation of H3K4 leads to DNA methylation and inhibition of rDNA transcription [56]. On the same line, JHDM1B, a human nucleolar demethylase of the same family (*i.e.* containing a JmjC domain), controls the repression of rDNA gene expression by specific demethylation of trimethylated H3K4, limiting cell growth and proliferation [57]. Therefore, a similar mechanism likely occurs in plants supporting the view that NOF1 is involved in a network of proteins acting through a chromatin-based regulation of rDNA transcription. In order to confirm this molecular role, it would be necessary to set up an inducible system *in planta* (e.g. RNAi) allowing to test the direct effect of switching off *NOF1* expression on H3K4 methylation and to search for JmjC demethylase homolog in Arabidopsis. Interestingly, a related JmjC-domain gene has been recently characterized, the mutation of which triggers ectopic cytosine methylation, probably through an increase in H3K9 methylation levels [58]. Last, it is worth to notice that the loss of JHD2 demethylase in yeast is not lethal, suggesting that the mutation of YIL091C would affect other nucleolar functions, in agreement with its interactions with several nucleolar proteins.

### A putative network of proteins involved in nucleolar functions

Among the proteins interacting with YIL091C are SAS10 and MPP10 [26], both involved in rRNA biogenesis. SAS10 plays a role in the structure of silenced chromatin [29], likely through H3 and H4 acetylation [59]. MPP10 is a part of the small subunit processome (SSU) required for rRNA biogenesis [14], [27], [28], [60]. Comparative genomics between Arabidopsis and yeast [61], [62] revealed a putative network of homologous proteins that could interact with NOF1 to control rRNA biogenesis (Figure S7). The genetic and functional characterizations of some of these proteins strongly support this hypothesis. For instance, SWA1 is a nucleolar protein, expressed in dividing cells, essential for female gametogenesis and involved in rRNA biogenesis [5]. TORMOZ (TOZ, At5g16750) is a nucleolar protein, required for cell division patterning, at least during embryo development (no null allele have been characterized), that may be also involved in rRNA biogenesis [7]. Therefore, it is tempting to speculate that a similar network of nucleolar proteins, including NOF1, is involved in the regulation of rRNA biogenesis in Arabidopsis. As in yeast, NOF1 and interacting proteins would constitue a molecular link between the regulation of rDNA gene expression and processing of rRNA, by filling the gap between the processome and the transcription of rDNA. Nevertheless, although coupling of rRNA biogenesis with cell growth has been established [12], [63], [64], [65], we cannot exclude that other nucleolar functions are affected by *NOF1* mutation (e.g. modification of small RNAs, assembling of ribonucleoproteins, or cell division). For instance, YIL091C was shown to interact with several kinases such as Swe1 that regulates transition from G2/M or Tos3, a tumor suppressor essential to mammalian embryo development [66].

## Materials and Methods

### Plant material, growth conditions and seed viability

*A. thaliana* seeds of the wild-type ecotype Wassilewskija (WS) as well as the primary transformants DKE14 and EXY42 lines were obtained from the IJPB seed stock center (INRA, Versailles, France, http://dbsgap.versailles.inra.fr/agrobactplus/English/Accueil_eng.jsp).Seeds were surface sterilized and germinated on Murashige and Skoog (MS) medium (M02 555, pH 5.6; Duchefa, Haarlem, the Netherlands) solidified with 0.7% (w/v) agar. After a cold treatment of 48 h at 4°C in the dark, the plates were transferred to a growth chamber and incubated at 20°C/15°C day/night, under a 16-h/8-h light/dark regime. Selection of T-DNA-containing seeds was performed by germination on MS supplemented with kanamycin (Sigma, Saint-Quentin Fallavier, France) at 50 mg l-1. After 15 days, the plantlets were transferred to sterilized compost in individual pots, grown under the same conditions as above and irrigated twice a week with a complete mineral nutrient solution. To analyze the distribution of seeds with phenotype (white and wrinkled seeds), hemizygous *nof1-1*/*NOF1-1* siliques at 15 DAF were opened and observed without disturbing seed positions. For time course studies, all the developing seeds of one shoot were harvested 3–4 weeks after the onset of flowering: siliques ranging from 3 to 22 DAF were opened and the corresponding seeds removed and sub-sampled. Material used for RNA extraction was immediately frozen in liquid nitrogen and stored at −80°C prior to extraction.

Viability tests were based on the reduction of tetrazolium salts to highly colored end products called formazans in viable seeds. Teguments of imbibed mutant and wild-type seeds were torn and embryos were soaked in a 1% 2,3,5-triphenyl tetrazolium chloride solution (Sigma, CA). Samples were incubated for 2 days in the dark at 30°C.

### DNA extraction and PCR analysis

RT–PCR experiments were performed as previously described [67]. All the oligonucleotides used in this study are described in table S3. Briefly, total RNA was extracted from different tissues using an RNA extraction kit (Mammalian total RNA extraction kit, SIGMA) supplemented with RNase-free Dnase (Qiagen, Germany) during the extraction. cDNAs were synthesized using the Superscript II (INVITROGEN) with (dT)_22_ according to the manufacturer's instructions. *NOF1* cDNA was amplified using Stock center cDNA C104805 with the primers B1dke14ATGgate and B1dke14STOPgate located at the start codon and 3′-end of the cDNA, respectively. For gene expression analysis, a 1449 bp fragment of the At1g17690 cDNA was amplified with the primers cDNA dkeUp (5′-GCACAGGTCCCATGAGAAATT-3′) and cDNA dkeLow (5′-TGTCAAAGGCAGGTGATTCCCA-3′). Controls were carried out with primers that amplify a constitutively expressed elongation factor ‘EF-1alpha’ cDNA as previously described [68].

### Intracellular localization of NOF1

The *NOF1* cDNA was amplified with the proofreading Pfu Ultra DNA polymerase (STRATAGENE, La Jolla, CA, USA) from cDNA obtained after reverse transcription of whole silique-extracted mRNAs using the *B1DKE14ATGgate* and *B2DKE14STPgate* oligonucleotides. The PCR product was introduced by a BP recombination into *pDONR207* entry vector (INVITROGEN, Carlsbad, CA, USA) and transferred into the binary vector pMDC83 vector [69] by a LR recombination reaction, to obtain a translational fusion between the *NOF1* and the *GFP* (C-terminal) reporter gene. This plasmid was used for stable as well as transient expression of NOF1:GFP.

### Genetic complementation of the *nof1* mutants

The *NOF1* genomic sequence (7218 bp length, containing 1500 bp of promoter sequence and 500 pb of 3′ sequence) was PCR amplified from DNA of the BAC F11A6 and blunt cloned into TOPOblunt vector (Invitrogen Carlsbad, California, USA). A XhoI/KpnI fragment was then subcloned into the KpnI/SalI restricted pBIB-HYG vector [70]. The resulting plasmid was introduced into Agrobacterium tumefaciens strain C58C1 pMP90 [71] by electroporation. Hemizygous plant *NOF1/nof1*-1 were transformed by infiltration [72], using surfactant Silwet L-77. Transformants were selected by growing seedlings on hygromycin (50 mg/ml). On the 123 primary transformants obtained, 18 were homozygous for the mutation (*nof1-1)* as suggested by the resistance of their progenies to kanamycin and confirmed by genotyping by PCR (data not shown*)*. The complementation of *nof1-2* allele was obtained by crossing hemizygous plants with complemented homozygous *nof1-1* plants and selecting for homozygous *nof1-2/nof1-2* in their progenies.

### Functional analysis of the *NOF1* promoter

The *NOF1* promoter used *(Pro_AtNOF_:uidA*) corresponds to region −1500 to −1 bp relative to the translational start codon and was amplified with the proofreading Pfu Ultra DNA polymerase (STRATAGENE, La Jolla, CA, USA) from BACF11A6 using *B1DKE14up* and *B2DKE14low*, *att*B1 and *att*B2 referring to the corresponding Gateway recombination sequences. The PCR product was introduced by a BP recombination into *pDONR207* entry vector (INVITROGEN, Carlsbad, CA, USA) and transferred to the binary vector *pBI101-R1R2-GUS* (F. Divol, J.-C. Palauqui, and B. Dubreucq, unpublished data) by a LR recombination reaction, to obtain a transcriptional fusion between the *NOF1* promoter (*pNOF1*) and the *uidA* reporter gene. Arabidopsis transformation was carried out as described above. Ten transformants were selected on MS medium containing kanamycin (50 mg.l^−1^) and then transferred to soil for further characterization.

### Immunolocalization

For immunolocalizations, samples were fixed for 1 h in 4% (w/v) paraformaldehyde embedded, sectioned and treated as previously described [73]. Epitope demasking was carried out by incubating the slides in buffer citrate 10 mM, PH 6 (0.1 M sodium citrate, 0.1 M citric acid) in EZ- RETRIEVER (Biogenex, San Ramon USA). A commercial goat antibody against PIN1 (aP-20, Santa Cruz Biotechnology, ref sc-276163) was used at 1∶100. Secondary antibodies were purchased from Molecular Probe (Alexa-conjugated donkey anti goat).

### Imaging and pictures measurements

Light microscopy was carried out as previously described [74]. Briefly, samples were fixed with 4% paraformaldehyde and 5% dimethyl sulfoxide in 0.1 M phosphate buffer pH 7, dehydrated in acetone and included in resin (Technovit 7100 kit, Heraus Kulzer, Germany), following the manufacturer's instructions. Semi-thin sections (4–8 mum) were performed with a Jung RM 2055 microtome (Leica), stained with toluidin blue (1% w/v in 0.1 M phosphate buffer pH 7.2; Sigma, CA). For analyses using Nomarski optics, seeds were removed from siliques and cleared for 1 to 24 h in a chloralhydrate solution (chloralhydrate-H2O-glycerol, 8∶2∶1, w:v:v) on a microscope glass slide. Samples were examined using an Axioplan II microscope (Zeiss, Jena, Germany) microscope with or without Nomarski optics. Photographs were taken using a progressC10 digital camera (Jenoptik, Jena, Germany). For confocal microscopy, A Leica TCS-SP2-AOBS spectral confocal lazer-scanning microscope (Leica Microsystems, Mannheim, Germany) was used. The excitation wavelength for DAPI, GFP and Alexa488 stained samples was 405, 488 nm, respectively emission was collected at 420 to 470 nm and 500–550 nm respectively. Samples for modified pseudo-Schiff propidium iodide staining procedure were prepared and imaged as recently described [75]. Data were processed for 3D volume rendering or 2D orthogonal sections using the open source software Osirix (http://homepage.mac.com/rossetantoine/osirix/) on a quadxeon 2,66 Ghz 2 GB RAM Apple Mac pro workstation. RGB stacks of confocal images were imported as DICOm files into Osirix prior to treatments.

### DNA methylation experiments

Genomic DNA was extracted from dissected seeds exhibiting in the same silique either the “white seed” mutant phenotype (*nof*1-1) or wild-type green seeds, using DNA extraction columns (DNeasy plant mini, Qiagen, Courtaboeuf, France). Thus the green seeds are called “hemi” since they contain 2/3 of heterozygous seeds (*nof 1-1/NOF1*) and 1/3 of WT seeds. DNA was PCR amplified using specific primers (p2f GCATGCAAAAAGAATTTTCA and p2r CTGGAAAAAGGCAACAAAAC) directly or after restriction with a methylation sensitive enzyme (*Hpa*II NEB Ipswich USA), following the manufacturer's instructions. The oligonucleotides were designed to amplify a genomic fragment including the transcription start signal and containing 6 *Hpa*II restriction sites. The amplification of the 25S rDNA using oligo nucleotides u7 and u8 was used as internal control on both genomic DNA templates since this fragment does not contain any *Hpa*II restriction site. The level of amplification is presented as a percentage of the internal standard *EF1alpha* gene (that contains no *Hpa*II site), to normalize genomic DNA variations between samples. Then a ratio between digested *versus* non-digested genomic DNA samples is calculated. Data presented are representative of 2 independent biological repeats and the error bars show the variations of three technical repeats).

## Supporting Information

Figure S1 Embryo phenotypes. A) Phenotypes of *nof1-1* embryos. Seeds were dissected after 1 hour of imbibition on whatman paper. Development ranges from globular (1) to almost fully shaped (2) embryos. B) Embryo viability using tetrazolium test (Boisson et al. 2001). Results shown are obtained with WT embryo (b) *nof1-1 embryo* (c and d) and wild type embryo boiled for 30 min as negative control (a). Bar  = 100 µM.
(2.77 MB PDF)

Figure S2 Phenotypes of *nof1-2* ovules. Siliques were dissected and cleared for DIC observations. A) row of developing ovules and B) an enlargement of the nuclei. Nuclei c–d and e exhibit typical figures of fertilized ovules whereas ovules a and b are blocked at the 4 nuclei stage of the megagametogenesis. Bar  = 10 µm.
(2.35 MB PDF)

Figure S3 Cytological analysis of *nof 1-1* embryos. A) 3D reconstructions of young embryos show defaults in cell divisions as figured onto (c) when compared to WT (a). Globular embryos show division abnormalities in the hypophysis (d,e, f) as well as in the suspensor cells, both in transverse and lengthways orientations. Bar  = 10 µM B) Mature dry seeds observed using confocal scanning microscopy after modified pseudo-Shiff propidium iodide staining (a–h). Several defects are typically found in almost fully shaped *nof 1-1* embryos when compared to WT (a): apical meristem is abnormal (d, arrow), ectopic divisions are found in the hypocotyl (e, f) or in the meristematic region (b) as well as defaults in cell adhesion (c). The quiecent center in the root meristem (arrow) display ectopic divisions and abnormal cellular organisation (g, WT and h, *nof 1-1*). Bars  = 20 µM (a, d, e, f), 15 µM (b, g, h), and 5 µM (c).
(5.06 MB PDF)

Figure S4 Complementation of the *nof1* mutations. The presence of the *nof1-1* or *nof1-2* T-DNA insertions was demonstrated in the progenies of transgenic seedlings by PCR using specific primers for *nof1-1* or nof1-2 insertions (Up/RB and 2821/LB3, respectively). Kanamycin resistance of the seedlings is provided by the nof1 mutations (see table S4) and hygromycin resistance by the new T-DNA carrying a functional copy of *NOF1* (see material and methods). Plant 6A2 is homozygous for *nof1-1*, 10B1 is homozygous for *nof1-2* and 17B3 carries both alleles. The complementation of homozygous plants for *nof1* mutations confirmed that *NOF1* mutations are responsible for the abnormal *nof1-1* and *nof1-2* phenotypes.
(0.03 MB PDF)

Figure S5 *NOF1* expression pattern. Electronic pictographic representations of *NOF1* expression patterns. Data analysis was performed using the the tools of the Bio-Array Resource at http://bar.utoronto.ca. (Winter et al., 2007).
(0.16 MB PDF)

Figure S6 Co-expression Analyses. Functional classification of genes according to MIPS database. A) best 100 genes co-expressed with *NOF1* (input set N = 102, classified set N = 99, Atgene express tissue set) B) random list of Arabidopsis genes (input set N = 100, classified set N = 99) C) random list of Arabidopsis genes (input set N = 1000, classified set N = 976) Data analysis was performed using the tools of the Bio-Array Resource at http://bar.utoronto.ca. (Provart and Zhu, 2003) and the Classification superviewer software (http://bbc.botany.utoronto.ca/ntools/cgi-bin/ntools classification_superviewer.cgi).
(0.06 MB PDF)

Figure S7 Predicted network of NOF1 (At1g17690) partners. The network was build using the interaction viewer program http://bar.utoronto.ca/interactions/cgi-bin/arabidopsis_interactions_viewer.cgi. (Toufighi et al., 2005). At5g16750 Toz (TORMOZ), At2g43650 EMB2777(SAS10), At2G47990 EDA13 (SWA1 Slow Walker, UTP15), At5g66540 AtMPP10, At5g15750 AtIMP4, At4g25630 FIB2 (Fibrillarin 2), At2g41500 Emb2776 (LIS Lachesis, a WD 40 small nuclear ribonucleoprotein).
(0.21 MB PDF)

Table S1 Segregation of the Kanamycin marker in the progenies of *nof* mutants. Cytological analyses suggesting that the mutations are embryo lethal, this hypothesis has been tested (H0 =  the segregation is 2Kr/1Ks). In both cases, the observed X2> X2 theoritical at 5% (3,84). The data showed that the number of resistant seedlings is lower than expected for embryo lethal mutations (more especially for the null allele *nof1-2*). This suggested that the transmission of the mutated gametes was affected. Therefore, the hypothesis of a gametophityc lethal mutation has been tested for the null allele *nof1-2* (expected ratio of 1Kr/1Ks). Again the hypothesis is rejected, the observed X2 was higher than expected. The data suggested in this case that probably the two type of gametes were affected. This hypothesis was confirmed by the analyses of reciprocal crosses (see Table S2).
(0.03 MB PDF)

Table S2 Reciprocal crosses between hemizygous *nof1* and WT plants: occurrence of embryo lethal phenotype and segregation of the Kanamycin resistance marker. A–B–C–D: Controls. Crossing hemizygous mutants with the wild type plants, no embryo phenotypes are expected. The observed dead seeds are naturally aborted seeds usually found in wild-type siliques and/or due to manual fertilization. E–F–G–H We wished to test if the transmission of the mutated gametes is affected or not. The hypothesis Ho  =  the transmission is not affected or the segregation ration is 1Kr/1 Ks‚ was tested. X2 cut off value is 3,84 at 5% risk. For *nof1-1* (E–F), the hypothesis is accepted at 5% risk, suggesting that there was no significant effect of the transmission of *nof1-1* gametes. For the null allele, *nof1-2*, the hypothesis is clearly rejected in both cases (G and H), suggesting that both types of gametes were affected. In addition, the lack of female gamete transmission demonstrated that the mutation is female gametophytic lethal.
(0.06 MB PDF)

Table S3 Oligonucleotides.
(0.02 MB PDF)
