# Supplementary figures and images for: NOF1 Encodes an Arabidopsis Protein Involved in the Control of rRNA Expression
Source: PLoS One. 2010 Sep 20;5(9):e12829. doi: 10.1371/journal.pone.0012829 (PMC2942902; doi:10.1371/journal.pone.0012829)

**A**

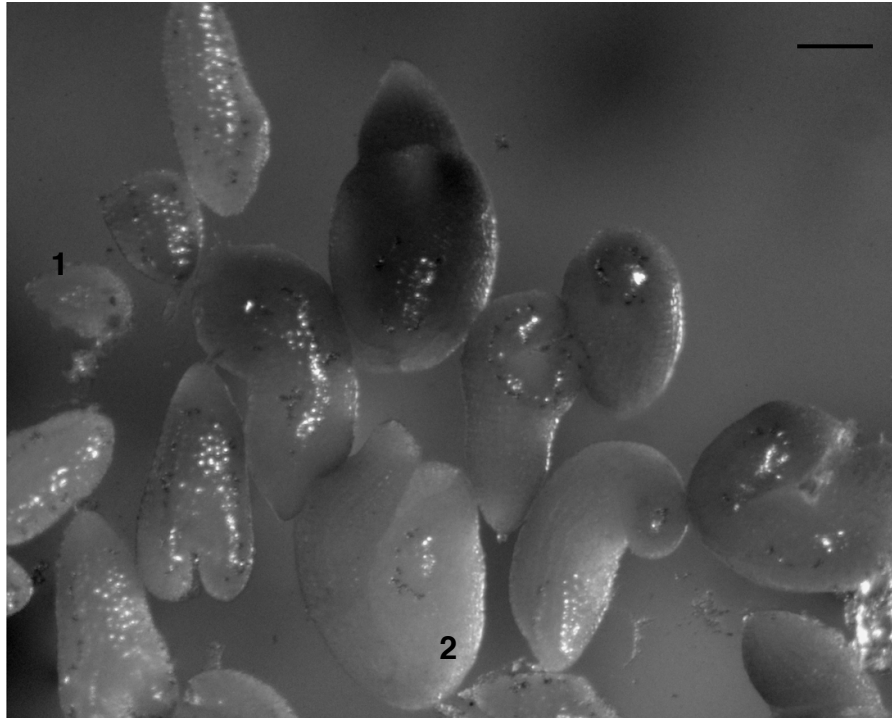

**B**

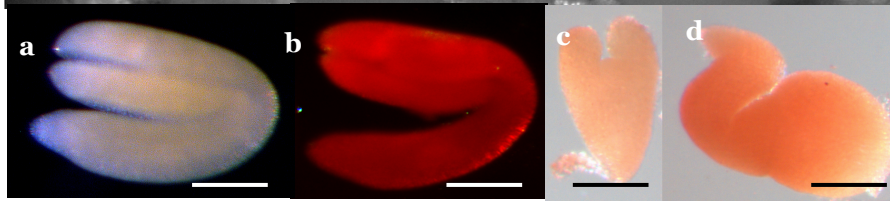

Supplement: Figure S1 — Embryo phenotypes. A) Phenotypes of nof1-1 embryos. Seeds were dissected after 1 hour of imbibition on whatman paper. Development ranges from globular (1) to almost fully shaped (2) embryos. B) Embryo viability using tetrazolium test (Boisson et al. 2001). Results shown are obtained with WT embryo (b) nof1-1 embryo (c and d) and wild type embryo boiled for 30 min as negative control (a). Bar = 100 µM. (2.77 MB PDF) [file pone.0012829.s001.pdf]

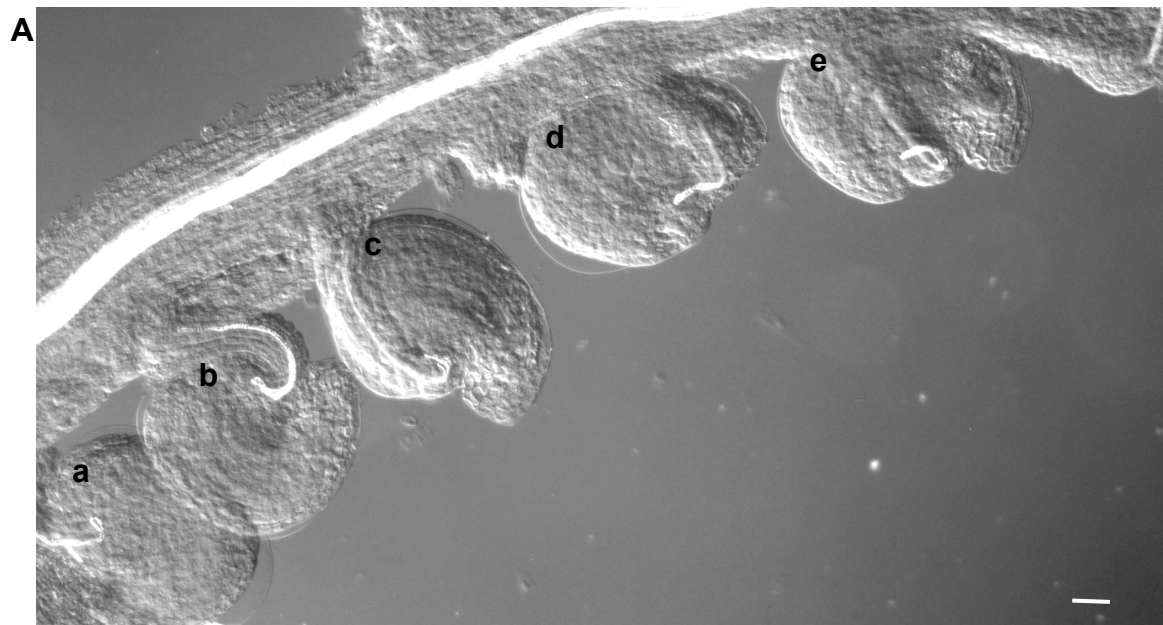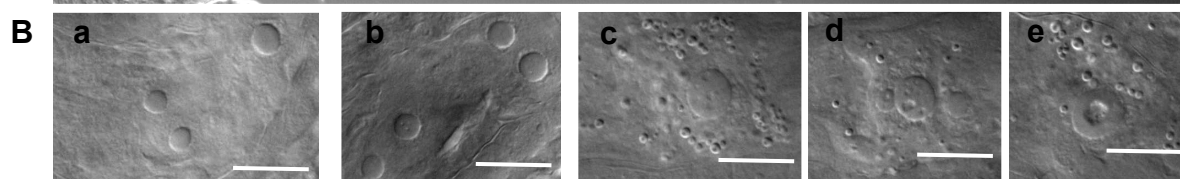

Supplement: Figure S2 — Phenotypes of nof1-2 ovules. Siliques were dissected and cleared for DIC observations. A) row of developing ovules and B) an enlargement of the nuclei. Nuclei c–d and e exhibit typical figures of fertilized ovules whereas ovules a and b are blocked at the 4 nuclei stage of the megagametogenesis. Bar = 10 µm. (2.35 MB PDF) [file pone.0012829.s002.pdf]

A

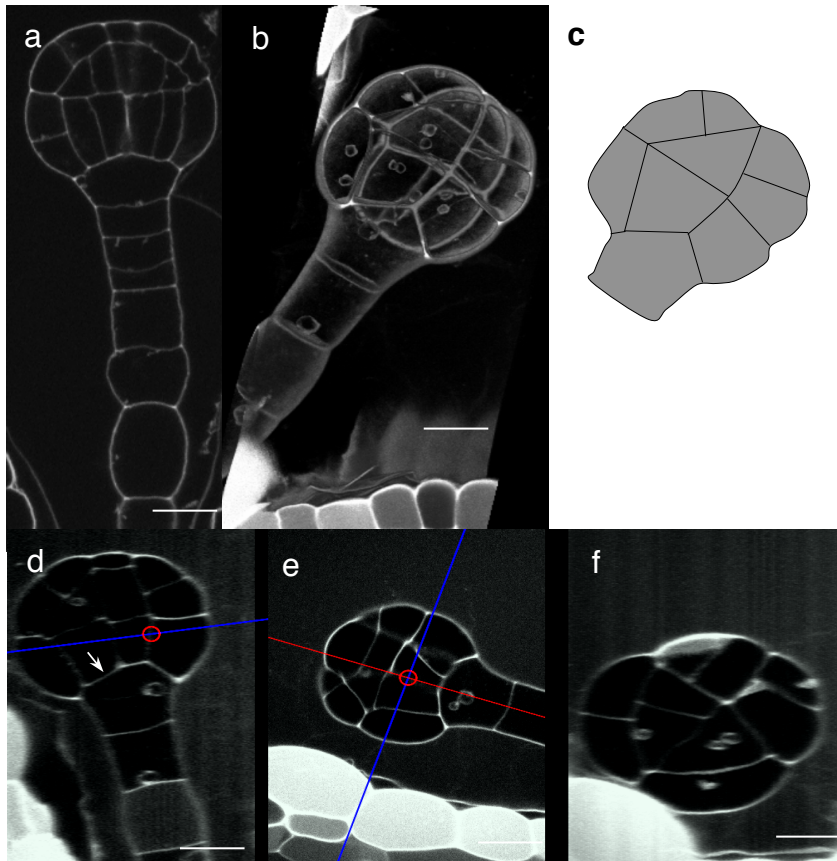

B

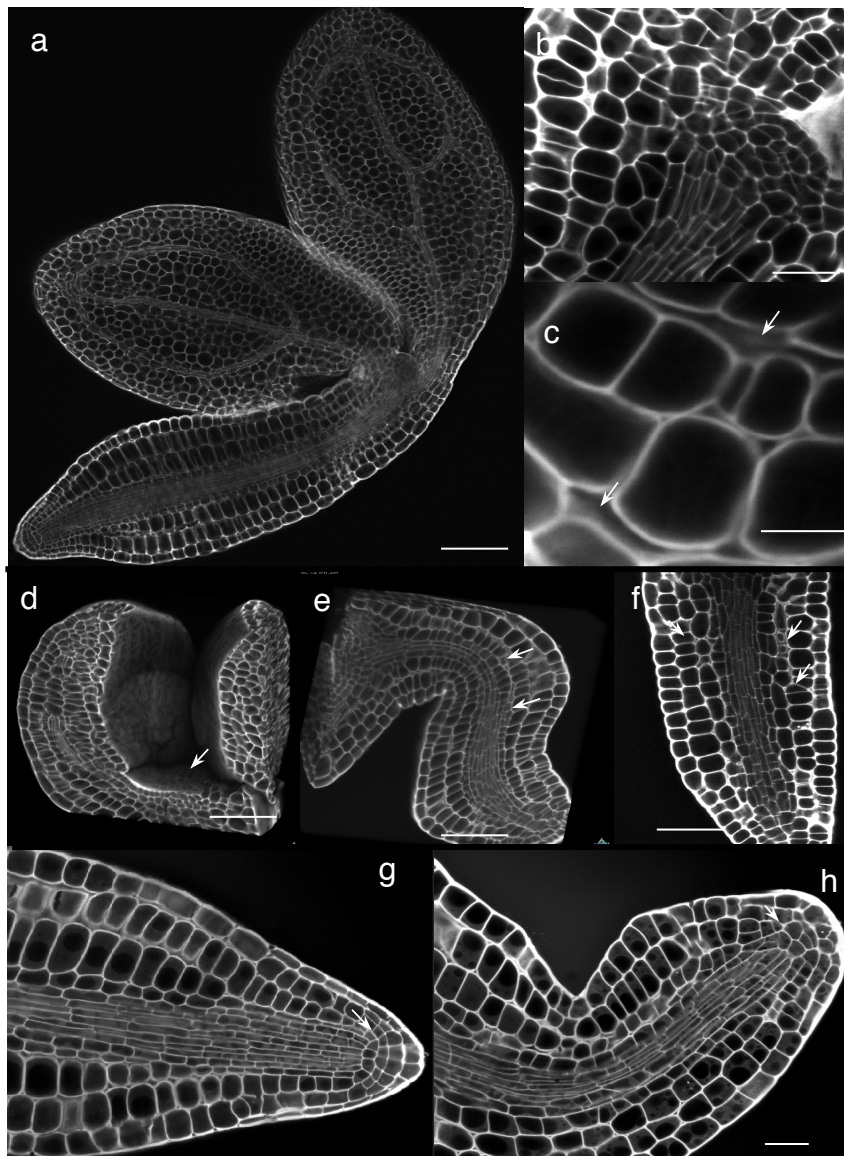

Supplement: Figure S3 — Cytological analysis of nof 1-1 embryos. A) 3D reconstructions of young embryos show defaults in cell divisions as figured onto (c) when compared to WT (a). Globular embryos show division abnormalities in the hypophysis (d,e, f) as well as in the suspensor cells, both in transverse and lengthways orientations. Bar = 10 µM B) Mature dry seeds observed using confocal scanning microscopy after modified pseudo-Shiff propidium iodide staining (a–h). Several defects are typically found in almost fully shaped nof 1-1 embryos when compared to WT (a): apical meristem is abnormal (d, arrow), ectopic divisions are found in the hypocotyl (e, f) or in the meristematic region (b) as well as defaults in cell adhesion (c). The quiecent center in the root meristem (arrow) display ectopic divisions and abnormal cellular organisation (g, WT and h, nof 1-1). Bars = 20 µM (a, d, e, f), 15 µM (b, g, h), and 5 µM (c). (5.06 MB PDF) [file pone.0012829.s003.pdf]

|                      | Up/RB<br>2821/LB3                                                                   | K <sup>r</sup> % | H <sup>r</sup> % |
|----------------------|-------------------------------------------------------------------------------------|------------------|------------------|
| <i>nof1-1/NOF1-1</i> | 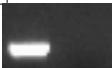   | 64               | 0                |
| <i>nof1-2/NOF1-2</i> | 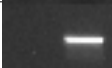   | 29               | 0                |
| Ws                   | 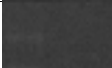  | 0                | 0                |
| 6A2                  | 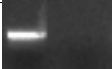 | 100              | 100              |
| 10B1                 | 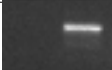 | 100              | 100              |
| 17B3                 | 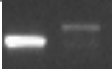 | 100              | 100              |

Supplement: Figure S4 — Complementation of the nof1 mutations. The presence of the nof1-1 or nof1-2 T-DNA insertions was demonstrated in the progenies of transgenic seedlings by PCR using specific primers for nof1-1 or nof1-2 insertions (Up/RB and 2821/LB3, respectively). Kanamycin resistance of the seedlings is provided by the nof1 mutations (see table S4) and hygromycin resistance by the new T-DNA carrying a functional copy of NOF1 (see material and methods). Plant 6A2 is homozygous for nof1-1, 10B1 is homozygous for nof1-2 and 17B3 carries both alleles. The complementation of homozygous plants for nof1 mutations confirmed that NOF1 mutations are responsible for the abnormal nof1-1 and nof1-2 phenotypes. (0.03 MB PDF) [file pone.0012829.s004.pdf]

A

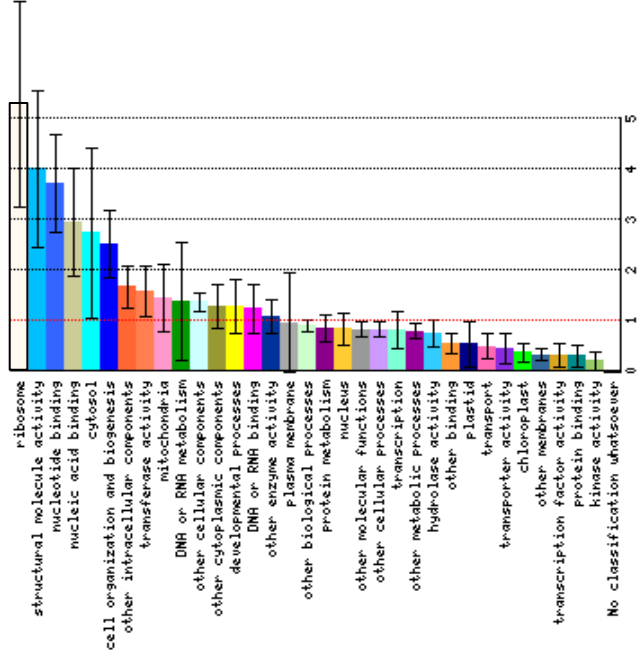

B

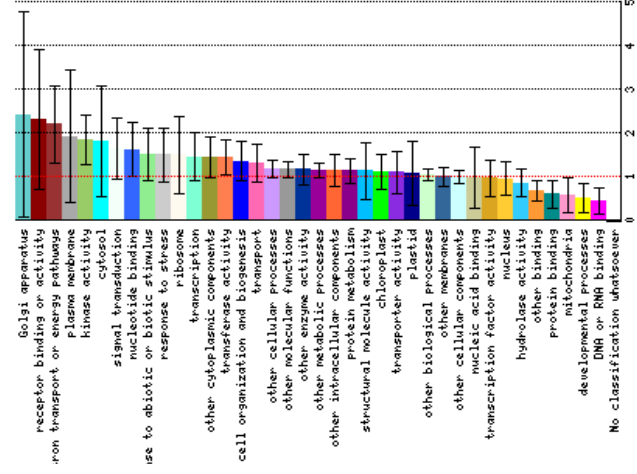

C

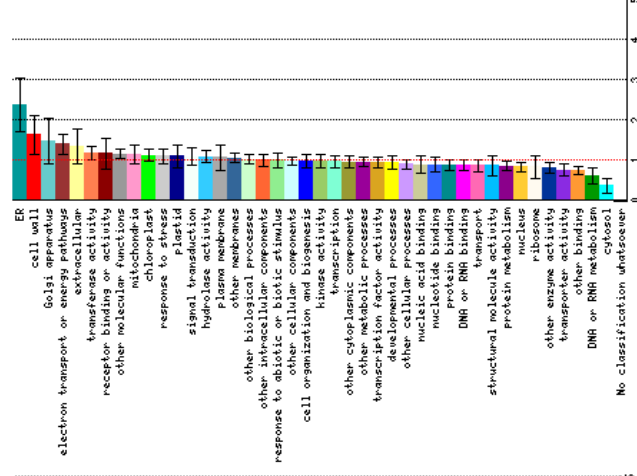

Supplement: Figure S6 — Co-expression Analyses. Functional classification of genes according to MIPS database. A) best 100 genes co-expressed with NOF1 (input set N = 102, classified set N = 99, Atgene express tissue set) B) random list of Arabidopsis genes (input set N = 100, classified set N = 99) C) random list of Arabidopsis genes (input set N = 1000, classified set N = 976) Data analysis was performed using the tools of the Bio-Array Resource at http://bar.utoronto.ca. (Provart and Zhu, 2003) and the Classification superviewer software (http://bbc.botany.utoronto.ca/ntools/cgi-bin/ntools classification_superviewer.cgi). (0.06 MB PDF) [file pone.0012829.s006.pdf]
