# Supplementary material for: NOF1 Encodes an Arabidopsis Protein Involved in the Control of rRNA Expression
Source: PLoS One. 2010 Sep 20;5(9):e12829. doi: 10.1371/journal.pone.0012829 (PMC2942902; doi:10.1371/journal.pone.0012829)
Supplement: Figure S5 — NOF1 expression pattern. Electronic pictographic representations of NOF1 expression patterns. Data analysis was performed using the the tools of the Bio-Array Resource at http://bar.utoronto.ca. (Winter et al., 2007). (0.16 MB PDF) [file pone.0012829.s005.pdf]

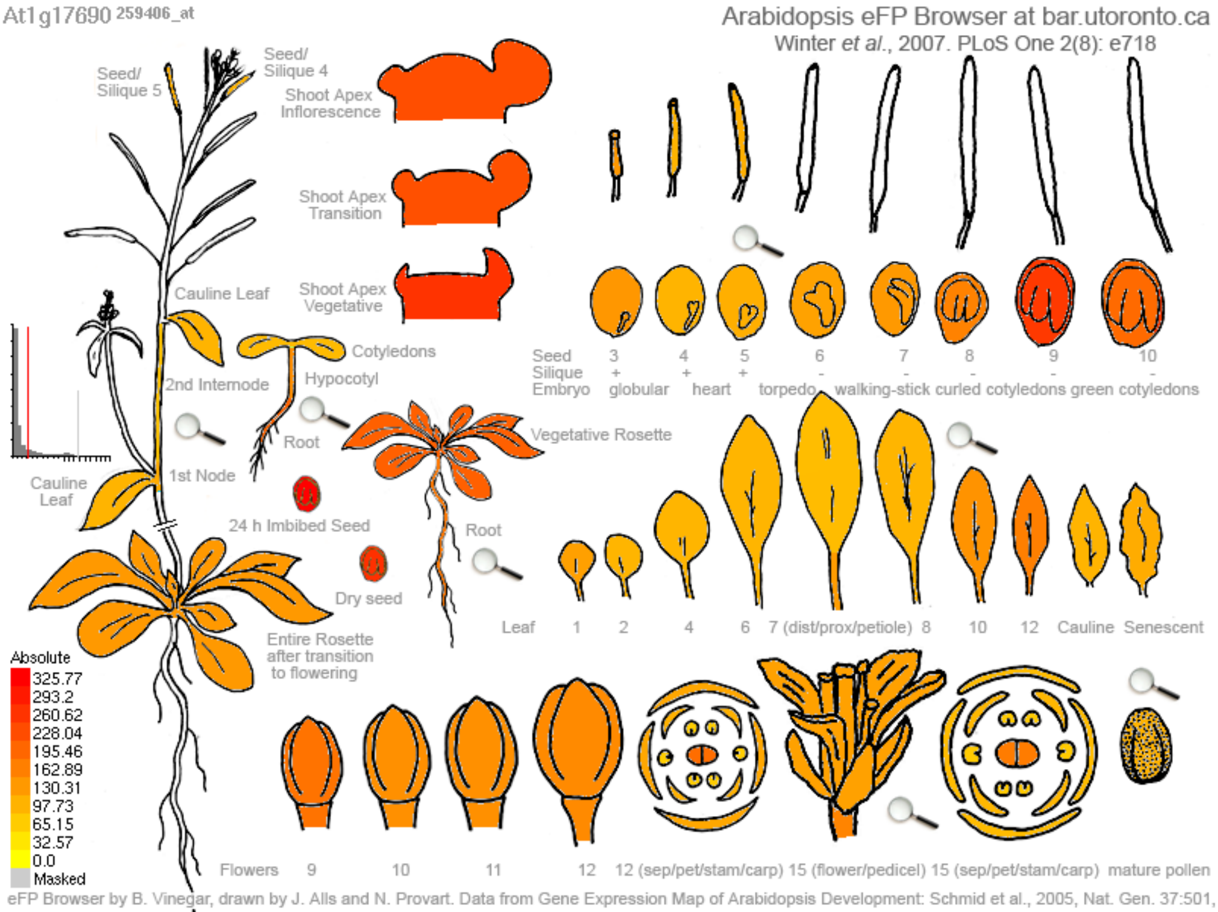

eFP Browser by B. Vinegar, drawn by J. Alls and N. Provart. Data from Gene Expression Map of Arabidopsis Development: Schmid et al., 2005, Nat. Gen. 37:501, and the Nambara lab for the imbibed and dry seed stages. Data are normalized by the GCOS method, TGT value of 100. Most tissues were sampled in triplicate.
