# Supplementary material for: NOF1 Encodes an Arabidopsis Protein Involved in the Control of rRNA Expression
Source: PLoS One. 2010 Sep 20;5(9):e12829. doi: 10.1371/journal.pone.0012829 (PMC2942902; doi:10.1371/journal.pone.0012829)
Supplement: Figure S7 — Predicted network of NOF1 (At1g17690) partners. The network was build using the interaction viewer program http://bar.utoronto.ca/interactions/cgi-bin/arabidopsis_interactions_viewer.cgi. (Toufighi et al., 2005). At5g16750 Toz (TORMOZ), At2g43650 EMB2777(SAS10), At2G47990 EDA13 (SWA1 Slow Walker, UTP15), At5g66540 AtMPP10, At5g15750 AtIMP4, At4g25630 FIB2 (Fibrillarin 2), At2g41500 Emb2776 (LIS Lachesis, a WD 40 small nuclear ribonucleoprotein). (0.21 MB PDF) [file pone.0012829.s007.pdf]

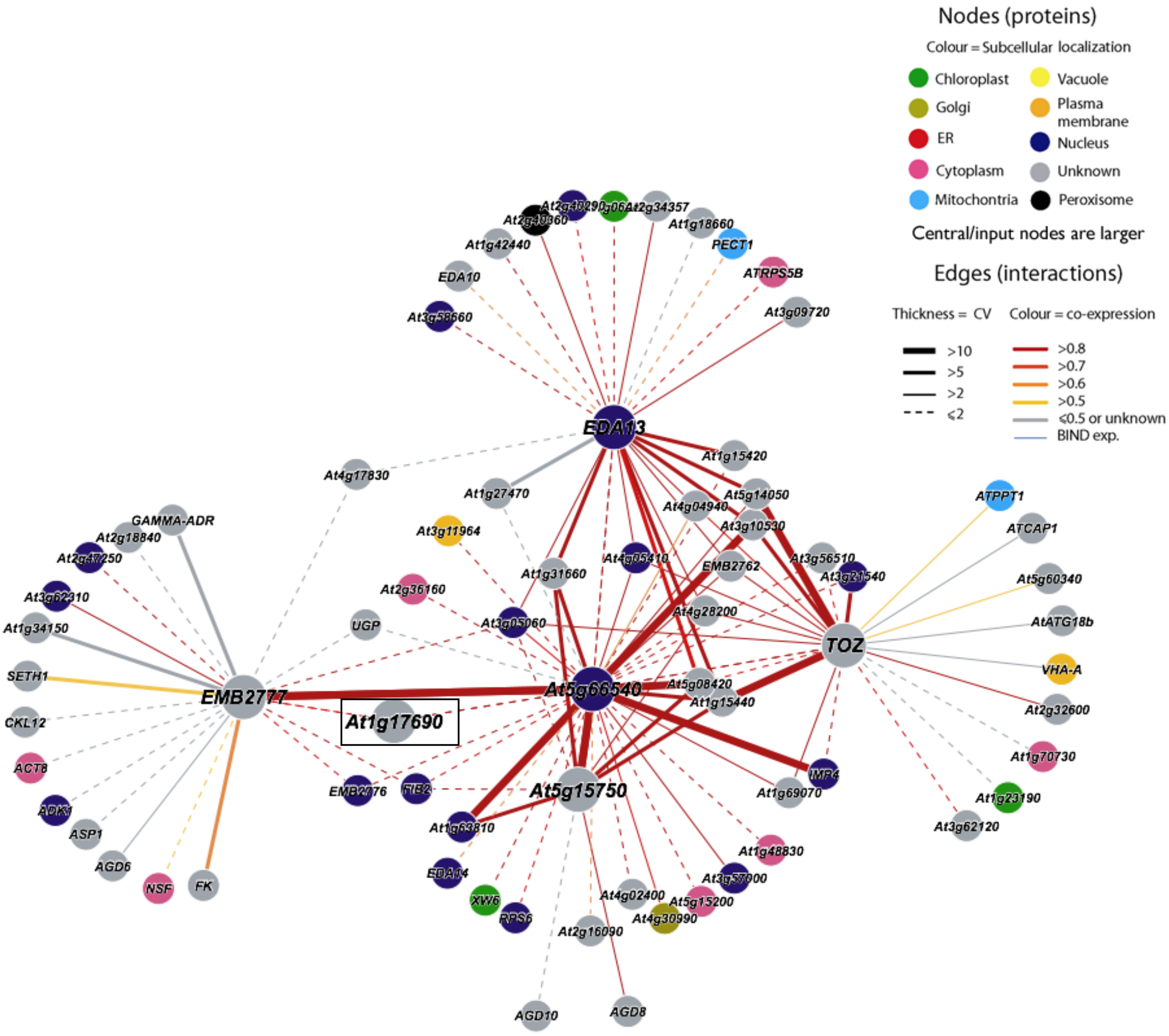

Nodes (proteins)
Colour = Subcellular localization
Chloroplast
Vacuole
Golgi
Plasma membrane
ER
Nucleus
Cytoplasm
Unknown
Mitochontria
Peroxisome
Central/input nodes are larger
Edges (interactions)
Thickness = CV
Colour = co-expression
>10
>5
>2
≤2
>0.8
>0.7
>0.6
>0.5
≤0.5 or unknown
BIND exp.
EDA13
At5g66540
At5g15750
EMB2777
TOZ
At1g17690
At1g42440
At1g18660
PECT1
ATRPS5B
At3g09720
EDA10
At3g58660
At2g34357
At4g17830
At1g27470
At1g15420
At5g14050
At4g04940
At3g10530
At3g11964
At1g31660
At4g05410
EMB2762
At3g56510
At3g21540
At2g36160
At3g05060
At4g28200
UGP
At5g08420
At1g15440
GAMMA-ADR
At2g18840
At2g47250
At3g62310
At1g34150
SETH1
CKL12
ACT8
ADK1
ASP1
AGD6
NSF
FK
EMB2776
FIB2
At1g63810
EDA14
XW6
RPS6
At2g16090
At4g02400
At4g30990
At5g15200
At3g57000
At1g48830
At1g69070
IMP4
AGD10
AGD8
ATPPT1
ATCAP1
At5g60340
AtATG18b
VHA-A
At2g32600
At1g70730
At1g23190
At3g62120
