# Supplementary material for: NOF1 Encodes an Arabidopsis Protein Involved in the Control of rRNA Expression
Source: PLoS One. 2010 Sep 20;5(9):e12829. doi: 10.1371/journal.pone.0012829 (PMC2942902; doi:10.1371/journal.pone.0012829)
Supplement: Table S1 — Segregation of the Kanamycin marker in the progenies of nof mutants. Cytological analyses suggesting that the mutations are embryo lethal, this hypothesis has been tested (H0 = the segregation is 2Kr/1Ks). In both cases, the observed X2> X2 theoritical at 5% (3,84). The data showed that the number of resistant seedlings is lower than expected for embryo lethal mutations (more especially for the null allele nof1-2). This suggested that the transmission of the mutated gametes was affected. Therefore, the hypothesis of a gametophityc lethal mutation has been tested for the null allele nof1-2 (expected ratio of 1Kr/1Ks). Again the hypothesis is rejected, the observed X2 was higher than expected. The data suggested in this case that probably the two type of gametes were affected. This hypothesis was confirmed by the analyses of reciprocal crosses (see Table S2). (0.03 MB PDF) [file pone.0012829.s008.pdf]

A

nof1-1/NOF1-1 self pollination	Kr	Ks	Total number of plants
Number of plants	1502	859	2361
Theoritical values	2/3	1/3	
Theoritical number	1574	787	$\chi^2 =$ 9,88

B

nof1-2/NOF1-2 self pollination	Kr	Ks	Total number of plants
Number of plants	268	647	915
Theoritical values	1/2	1/2	
Theoritical number	457,5	457,5	$\chi^2 =$ 142,8
