# Supplementary material for: NOF1 Encodes an Arabidopsis Protein Involved in the Control of rRNA Expression
Source: PLoS One. 2010 Sep 20;5(9):e12829. doi: 10.1371/journal.pone.0012829 (PMC2942902; doi:10.1371/journal.pone.0012829)
Supplement: Table S2 — Reciprocal crosses between hemizygous nof1 and WT plants: occurrence of embryo lethal phenotype and segregation of the Kanamycin resistance marker. A–B–C–D: Controls. Crossing hemizygous mutants with the wild type plants, no embryo phenotypes are expected. The observed dead seeds are naturally aborted seeds usually found in wild-type siliques and/or due to manual fertilization. E–F–G–H We wished to test if the transmission of the mutated gametes is affected or not. The hypothesis Ho = the transmission is not affected or the segregation ration is 1Kr/1 Ks‚ was tested. X2 cut off value is 3,84 at 5% risk. For nof1-1 (E–F), the hypothesis is accepted at 5% risk, suggesting that there was no significant effect of the transmission of nof1-1 gametes. For the null allele, nof1-2, the hypothesis is clearly rejected in both cases (G and H), suggesting that both types of gametes were affected. In addition, the lack of female gamete transmission demonstrated that the mutation is female gametophytic lethal. (0.06 MB PDF) [file pone.0012829.s009.pdf]

A

♀ *nof1-1/NOF1-1*<br>♂ WT	[emb] seeds	[WT] seeds	total seeds number
observed	4	102	106
expected ratio	0%	100%	
expected	0	106	

B

♀ WT<br>♂ *nof1-1/NOF1-1*	[emb] seeds	[WT] seeds	Nb de graines totales
observed	4	239	243
expected ratio	0%	100%	
expected	0	243	

C

♀ *nof1-2/NOF1-2*<br>♂ WT	[emb] seeds	[WT] seeds	Nb de graines totales
observed	7	178	185
expected ratio	0%	100%	
expected	0	185	

D

♀ WT<br>♂ *nof1-2/NOF1-2*	[emb] seeds	[WT] seeds	Nb de graines totales
observed	9	465	474
expected ratio	0%	100%	
expected	0	474	

E

♀ *nof1-1/NOF1-1*<br>♂ WT	[Kr] plantlets	[Ks] plantlets	Total plant number
observed	39	52	91
expected ratio	1/2	1/2	
expected	45,5	45,5	χ^2= 1,86

F

♀ WT<br>♂ *nof1-1/NOF1-1*	[Kr] plantlets	[Ks] plantlets	Total plant number
observed	121	106	227
expected ratio	1/2	1/2	
expected	113,5	113,5	χ^2= 0,99

G

♀ *nof1-2/NOF1-2*<br>♂ WT	[Kr] plantlets	[Ks] plantlets	Total plant number
observed	0	89	89
expected ratio	1/2	1/2	
expected	44,5	44,5	χ^2= 89

H

♀ WT<br>♂ *nof1-2/NOF1-2*	[Kr] plantlets	[Ks] plantlets	Total plant number
observed	163	257	420
expected ratio	1/2	1/2	
expected	210	210	χ^2= 21,04
