# Supplementary material for: NOF1 Encodes an Arabidopsis Protein Involved in the Control of rRNA Expression
Source: PLoS One. 2010 Sep 20;5(9):e12829. doi: 10.1371/journal.pone.0012829 (PMC2942902; doi:10.1371/journal.pone.0012829)
Supplement: Table S3 — Oligonucleotides. (0.02 MB PDF) [file pone.0012829.s010.pdf]

B1dke14ATGgate	GGGGACAAGTTTGTACAAAAAAGCAGGCTGATGGCTCCAAACGCTACTG
B1dke14STOPgate	GGGGACCACTTTGTACAAGAAAGCTGGGTCACAGAAAGCAAACATGTTCT
B1DKE14up	GGGGACAAGTTTGTACAAAAAAGCAGGCTGGATCTTTTGTGTTGGTTGATGCCA
B2DKE14low	GGGGACCACTTTGTACAAGAAAGCTGGGTCCTTAGGGAAGTTAACTGGTG
EF1aA4UP	ATGCCCCAGGACATCGTGATTTCAT
EF1aA4Low	TTGGCGGCACCCTTAGCTGGATCA
U1	CGTAACGAAGATGTTCTTGGC
U2	TGCGACCCTTCCATAAGTC
U3	TGGAGCGATTTGTCTGGTTA
U4	CTGCAATTCACACCAAGTAT
18Sfor	TGACGGAGAATTAGGGTTC
18Srev	CCTCCAATGGATCCTCGTTA
5,8Sfor	GCAACGGATATCTCGCCTCTC
5,8Srev	TGCGTTCAAAGACTCGATGG
25SFfor	AACGGGCTTGGCAGAATCAG
25Srev	ACGGACTTAGCCAACGACAC
p2for	GCATGCCAAAAAGAATTTTCAA
p2rev	CTCGGAAAAAGGCAACAAAACC
Up	AGATGAACCAAGAGACACCAGCAAA
dke pg2821	GCAATGATAAAAGAAGTACCTCT
LB3	GACAACTTTCCTTCTCATCT
RB2	CTGATACCAGACGTTGCCCG
